# PI3K/mTORC2 regulates TGF-β/Activin signalling by modulating Smad2/3 activity via linker phosphorylation

**DOI:** 10.1038/ncomms8212

**Published:** 2015-05-22

**Authors:** Jason S. L. Yu, Thamil Selvee Ramasamy, Nick Murphy, Marie K. Holt, Rafal Czapiewski, Shi-Khai Wei, Wei Cui

**Affiliations:** 1 Department of Surgery and Cancer, Institute of Reproductive and Developmental Biology, Imperial College London, Du Cane Road, London W12 0NN, UK

## Abstract

Crosstalk between the phosphatidylinositol 3-kinase (PI3K) and the transforming growth factor-β signalling pathways play an important role in regulating many cellular functions. However, the molecular mechanisms underpinning this crosstalk remain unclear. Here, we report that PI3K signalling antagonizes the Activin-induced definitive endoderm (DE) differentiation of human embryonic stem cells by attenuating the duration of Smad2/3 activation via the mechanistic target of rapamycin complex 2 (mTORC2). Activation of mTORC2 regulates the phosphorylation of the Smad2/3-T220/T179 linker residue independent of Akt, CDK and Erk activity. This phosphorylation primes receptor-activated Smad2/3 for recruitment of the E3 ubiquitin ligase Nedd4L, which in turn leads to their degradation. Inhibition of PI3K/mTORC2 reduces this phosphorylation and increases the duration of Smad2/3 activity, promoting a more robust mesendoderm and endoderm differentiation. These findings present a new and direct crosstalk mechanism between these two pathways in which mTORC2 functions as a novel and critical mediator.

## Abstract (web summary)

Although crosstalk between the phosphatidylinositol 3-kinase (PI3K) and transforming growth factor-β pathways is important, the mechanism is obscure. Here, Yu *et al*. show that activation of mTORC2 downstream of PI3K leads to the linker phosphorylation of Smad2/3 and their ubiquitin-dependent degradation.

## Graphical abstract

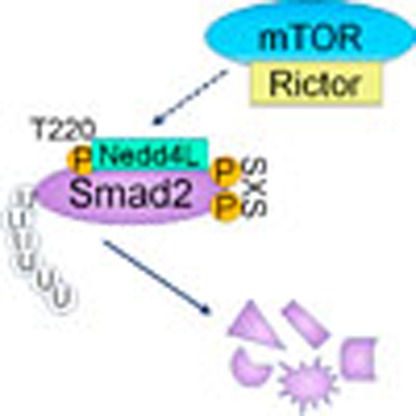

Cytokines of the transforming growth factor-β (TGF-β) superfamily including Nodal and Activin, control many cellular functions, such as cell growth, apoptosis and cell fate determination. These functions are not only controlled by the TGF-β pathway itself but are also extensively regulated by crosstalk between TGF-β and other signalling pathways^1^,^2^,^3^. TGF-β/Activin signalling is initiated upon ligand binding and activation of receptor complexes, which leads to the phosphorylation of the Smad2 and Smad3 (henceforth Smad2/3) C-terminal SxS motif, promoting their interaction with Smad4 and facilitating the translocation of the Smad2/3–Smad4 complexes to and accumulation within the nucleus where they regulate targeted gene expression in cooperation with other cofactors^4^,^5^,^6^. The efficacy of this pathway is not solely determined by the abundance of ligands and receptors but is also influenced by other signalling pathways^3^. Notably, the phosphatidylinositol 3-kinase (PI3K) pathway has been shown to alleviate TGF-β-induced apoptosis and cell cycle arrest in several tumour cell lines^7^,^8^,^9^,^10^, as well as inhibiting the Activin-induced DE differentiation of human embryonic stem cells (hESCs)^11^,^12^,^13^. However, the molecular mechanisms behind these effects remain contentious^8^,^9^,^10^. Although it has recently been proposed that the negative effects of PI3K upon DE differentiation are an indirect effect attributed to the inhibition of the Wnt-β-catenin pathway^14^, it is unclear how this mechanism results in enhanced Smad2/3 activity, thereby positing the existence of a more direct relationship between these two pathways^15^.

In this study, we demonstrate that PI3K signalling has a direct inhibitory effect on Activin-induced Smad2/3 activity in hESCs via the activation of mechanistic target of rapamycin complex 2 (mTORC2), leading to a reduction of Smad2/3 transcriptional activity and DE differentiation efficacy. PI3K/mTORC2 negatively regulates Smad2/3 activity by modulating their degradation via phosphorylation of a particular threonine residue within the Smad2/3 linker region. Our results therefore demonstrate a new and novel mechanism underpinning the crosstalk between the PI3K/mTOR and TGF-β/Activin signalling axes and in particular, firmly establishes mTORC2 as a critical mediator in modulating Smad2/3 activity.

## Results

### PI3K inhibits Activin-induced DE differentiation of hESCs

**Figure 1:**
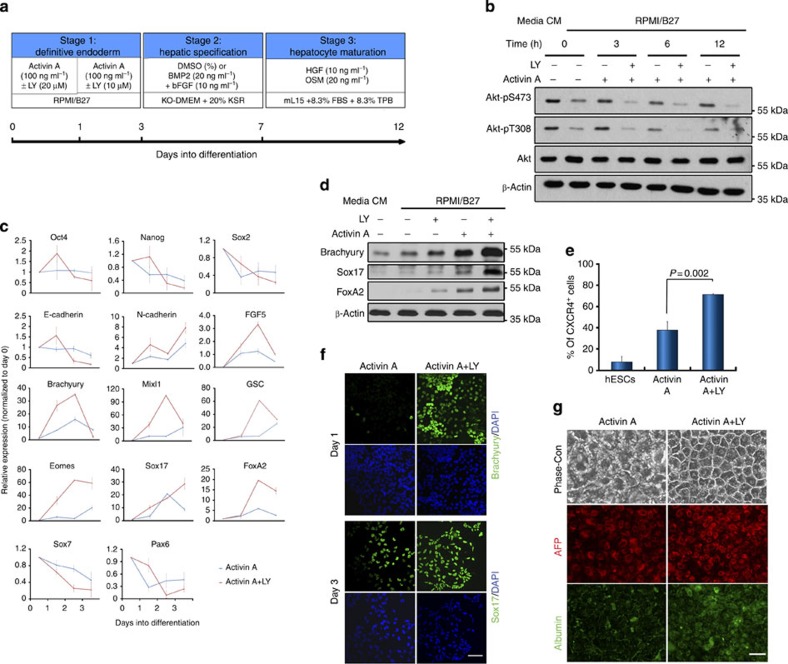
Inhibition of PI3K signalling promotes differentiation of hESCs to the definitive endoderm (DE). (**a**) Schematic illustrating the DE and hepatocyte differentiation protocol. (**b**) H1 hESCs cultured in MEF-CM (CM) were transferred into defined medium, RPMI/B27, for 1 h (time 0) prior to treatment with Activin A±LY294002 (LY). Cell lysates were collected at indicated time points and analysed by immunoblot. (**c**) Gene expression analysis by qRT–PCR in Activin A-treated hESCs with (red line) or without (blue line) LY. Data represent mean±s.d. from six measurements of two independent differentiations. (**d**) Immunoblot showing mesendoderm marker expression in hESCs treated for 24 h with indicated factors. (**e**) Percentage of CXCR4-positive cells by flow cytometry in hESCs with or without indicated treatment for 3 days. Data represent mean±s.d. from three independent biological samples. *P* value was calculated using the Student's *t*-test. (**f**) Immunostaining with Brachyury and Sox17 antibodies in hESCs treated as indicated. Scale bar, 50 μm. (**g**) hESCs were initially treated with Activin A±LY and then further differentiated to hepatocytes. Phase-contrast images (Phase-con) and immunostaining with AFP and albumin antibodies are presented. Scale bar, 50 μm.

To decipher the mechanisms underlying the antagonistic impact of the PI3K pathway upon TGF-β/Activin activities and the DE differentiation of hESCs, we developed a serum-free and chemically defined culture condition to convert hESCs to DE, in which high-dosage Activin A (henceforth AA) was shown to enhance the activation of Smad2/3 signalling and DE differentiation as previously reported (Supplementary Fig. 1a; Fig. 1a)^11^,^12^,^16^. Under this culture condition, treatment of hESCs with LY294002 (LY), a PI3K inhibitor, diminished Akt activation even in the presence of AA (Fig. 1b). In comparison with the differentiation using AA alone, co-treatment of hESCs with AA and LY evidently enhanced the Activin-induced DE differentiation as shown by a higher expression of mesendoderm and DE markers (Fig. 1c–f). This LY-dependent enhancement of DE differentiation was further corroborated by an increase in the generation of functional hepatocyte-like cells and in multiple hESC lines (Fig. 1g; Supplementary Fig. 1b,c). Therefore, this chemically defined culture system provides a useful platform from which to further interrogate the underlying molecular mechanisms driving the improvement of DE specification.

### Inhibiting PI3K prolongs Activin-induced Smad2/3 activation

**Figure 2:**
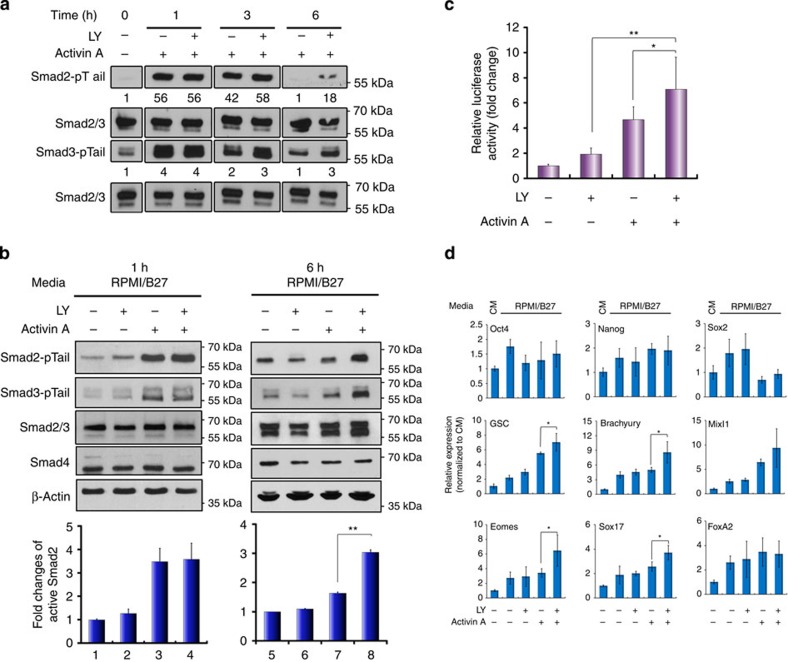
Suppression of PI3K signalling prolongs Activin-induced Smad2/3 activity. (**a**,**b**) hESCs were treated with Activin A or/and LY for the indicted time points before being subjected to immunoblotting analysis. Numbers in **a** represent the quantification of activated Smad2 and Smad3. Upper panels in **b** are representative immunoblot and lower panels are histograms of densitometric measurements from three independent biological samples. (**c**) Luciferase assay in hESCs co-transfected with pGL3-CAGA_12_-luc and renilla constructs and treated for 6 h. Data show mean±s.d. of three independent transfection experiments. (**d**) Messenger RNA expression of indicated markers by qRT–PCR. Bar graphs indicate fold induction. Data represent mean±s.d. of six measurements from two independent experiments. **P<0.001 and *P≤0.05 by the Student's *t*-test. LY, LY294002.

To interrogate the mechanisms through which PI3K signalling interferes with TGF-β activities, we first evaluated the activation status of Smad2/3 under AA±LY conditions. Time-course analysis revealed that although LY treatment had no effect on the initial activation of Smad2/3 (Smad2/3-pTail), the decline of active Smad2/3 was clearly attenuated in AA–LY-treated cells compared with cells treated with AA alone (Fig. 2a), resulting in a marked increase of active Smad2/3 six hours post treatment (Fig. 2b). AA–LY-treated cells also exhibited a significant increase in Smad2/3 transcriptional activity evidenced by higher luciferase reporter activity (Fig. 2c) and a considerable upregulation of mesendoderm markers MixL1, Eomes and goosecoid and DE marker Sox17, which are known Smad2/3 targets (Fig. 2d)^1^,^17^,^18^. This finding implies preferential specification of the cells towards the mesendoderm and DE even at this early stage of differentiation. Furthermore, to rule out the possibility that these enhancements were due to any off-target effects of LY, we treated hESCs with two other PI3K inhibitors in the place of LY, the more commonly used wortmannin and the more selective Pictilisib (also known as GDC-0941). Both experiments largely replicated the changes observed using AA–LY treatment (Supplementary Fig. 2) Therefore, inhibition of PI3K directly enhances Activin-induced DE formation by extending the duration of Smad2/3 activity.

### PI3K modulates ubiquitination and degradation of Smad2/3

Since inhibition of PI3K extended the duration of Smad2/3 activation rather than altering their initial response to AA, we reasoned that PI3K signalling might affect Smad2/3 activity by regulating its turnover, either via phosphatase-mediated dephosphorylation of the SxS motif or by ubiquitin-mediated proteasomal degradation of the active Smad2/3 protein^2^. Until recently, nuclear PPM1A was the only phosphatase identified to dephosphorylate Smad2/3 at the SxS motif^19^, which is inhibited by CLIC4 (chloride intracellular channel 4) protein^20^. However, not only did LY treatment not affect the expression of either protein, PPM1A was also detected primarily in the cytoplasm (Supplementary Fig. 3a,b). It is therefore unlikely that Smad2/3 dephosphorylation by PPM1A is the mechanism accounting for the phenotypic changes associated with AA–LY treatment. Although one recent study has shown that PP5, a member of the PPP phosphatase family, is able to dephosphorylate active Smad2/3 upon overexpression^21^, treatment of hESCs with okadaic acid, a potent inhibitor of PPP phosphatases, had no effect on the decay kinetics of active Smad2 (Supplementary Fig. 3c). Furthermore, enhanced TGF-β-induced transcriptional responses that were observed in PP5-null mice has been shown to result from increased levels of Smad3 protein rather than through any direct effect upon the duration of their activation^21^. These findings therefore further discount the involvement of phosphatases in our underlying mechanism.

**Figure 3:**
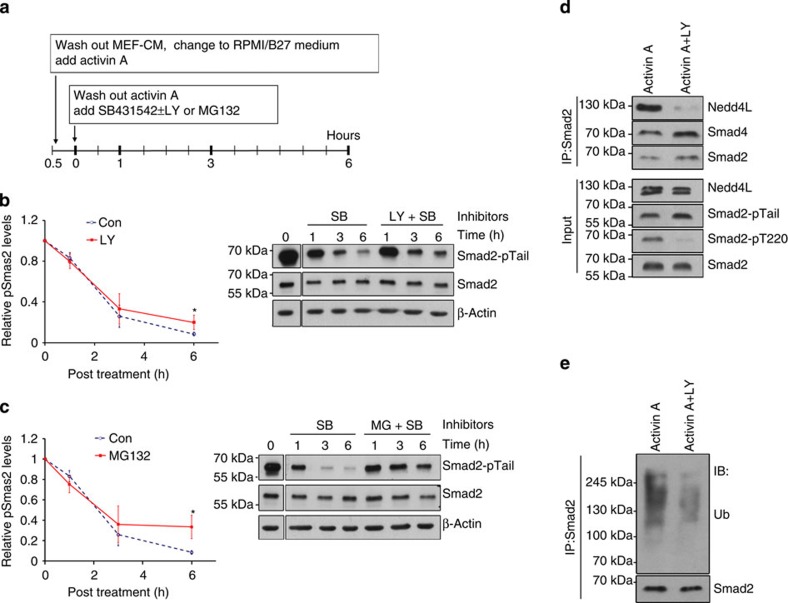
Inhibition of PI3K reduces Smad2/3 interaction with Nedd4L and subsequent ubiquitination. (**a**) Scheme of treatment for **b** and **c**. (**b**,**c**) Representative immunoblot (right) and quantifications (left) of activated Smad2 (Smad2-pTail) in hESCs treated as illustrated in **a** with SB±LY (**b**) or SB±MG132 (**c**). Graphs represent mean±s.d. from three independent experiments. (**d**,**e**) hESCs treated with Activin A±LY for 1 h were analysed by Smad2 co-immunoprecipitation with indicated antibodies. *P≤0.05 by the Student's *t*-test. SB, SB431542; LY, LY294002; MG, MG132; Ub, ubiquitin.

Given that ubiquitin-mediated proteasomal degradation is another mechanism by which Smad2/3 activity is terminated^22^, we next investigated whether LY acts to protect active Smad2 from proteasomal degradation. Similar decay kinetics for active Smad2 was observed between LY- and MG132-treated cells, albeit not with the same efficacy (Fig. 3a–c). In addition, inhibition of the Activin receptor by SB431542 did not abolish the effect of LY, but rather made it observable much earlier at 1 h post treatment (Fig. 3b). Together, this suggests that PI3K affects the TGF-β/Activin pathway independent of receptor activity and acts to promote the ubiquitin-mediated proteasomal degradation of active Smad2/3. Although Nedd4L has been previously identified as the ubiquitin ligase responsible for Smad2/3 ubiquitination^23^, we observed no LY-dependent changes in its expression (Supplementary Fig. 3d). However, LY treatment substantially reduced its interaction with Smad2 (Fig. 3d), which was corroborated by decreased ubiquitination of Smad2 (Fig. 3e). These results therefore highlight the critical role PI3K signalling plays in regulating the association of Nedd4L with Smad2/3, which in turn, dictates the duration and efficacy of Smad2/3 activity.

### PI3K primes Smad2/3 interaction with Nedd4L via pT220/T179

**Figure 4:**
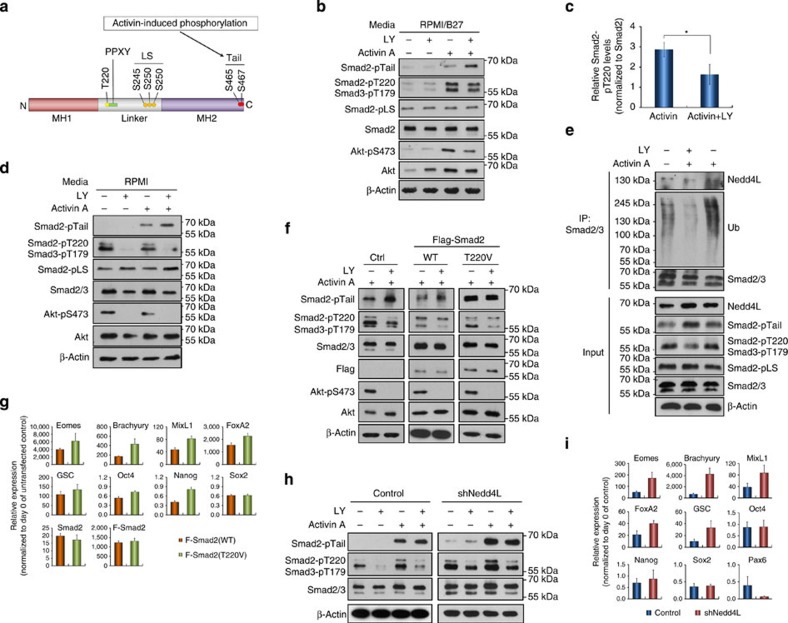
PI3K regulates Nedd4L-mediated Smad2/3 degradation via phosphorylation of the T220/T179 linker residue. (**a**) A schematic illustrating structural domains of Smad2 protein. The C-terminal and linker phosphorylation sites as well as the E3 ubiquitin ligase-binding PPXY motif are indicated. (**b**,**c**) Representative immunoblot (**b**) and quantification (**c**) showing significant reduction of Smad2-pT220 in hESCs at 6 h post treatment with Activin A+LY. Data in **c** represent mean±s.d. from three independent experiments. *P<0.05 (Student *t*-test). (**d**) Immunoblot showing that PC3 cells exhibited a similar pattern of Smad2 phosphorylation at various residues as that of hESCs in response to Activin±LY treatment after overnight starvation. (**e**) Nedd4L-mediated Smad2/3 ubiquitination requires phosphorylation at both linker T220/T179 and C-terminal SxS sites. PC3 cells were treated with Activin A±LY for 1 h, with MG132 added into the cultures 30 min prior to lysis. Smad2/3 immunoprecipitates were analysed by immunoblotting with indicated antibodies. (**f**) Immunoblot showing that Smad2-T220V mutant abolished the effect of LY on Activin-induced Smad2 activation. PC3 cells expressing Flag-tagged WT or T220V mutant (T220V) Smad2 were starved overnight, followed by treatment for 1 h with Activin A±LY. (**g**) qRT–PCR showing that hESCs expressing Smad2-T220V gave rise to higher levels of DE gene expression in response to Activin A. (**h**) Knockdown of Nedd4L in PC3 cells abolished the effect of LY on Activin-induced Smad2 activation. (**i**) qRT–PCR showing hESCs of Nedd4L knockdown exhibiting higher DE gene expression. hESCs in **g** and **i** were treated with Activin A for 2 days before the analysis, and data represent mean±s.d. from at least six measurements of two independent experiments.

**Figure 5:**
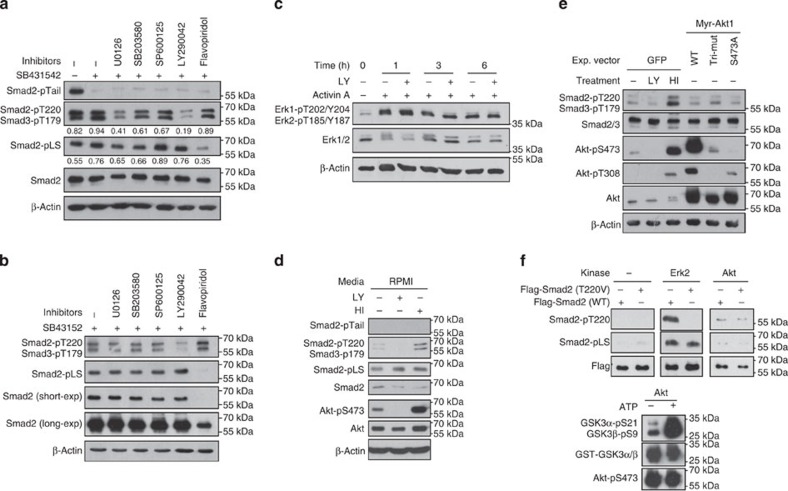
Phosphorylation of Smad2/3 linker residues are differentially regulated by various signalling pathways. (**a**,**b**) Immunoblot of Smad2/3 phosphorylation in hESCs that were pre-treated with Activin A, then incubated with indicated inhibitors for 1 h (**a**) or 6 h (**b**). (**c**) Immunoblot with Erk1/2 antibodies on cell extracts from hESCs with indicated treatments. (**d**) hESCs were starved for1 h in RPMI medium followed by treatment with either LY or HI for 1 h before being analysed by immunoblotting. (**e**) Hep3B cells transiently expressing either green fluorescent protein (GFP) or the various forms of Akt as indicated were starved overnight while GFP-expressing cells were treated with either LY or HI for 1 h before harvest for immunoblot. (**f**) *In vitro* kinase assay. Active Akt was isolated from PC3 cells by immunoprecipitation and incubated with recombinant GST-Flag-tagged wild-type (WT) or T220V mutant Smad2 in the presence of ATP. The protein mixtures were then resolved by SDS–polyacrylamide gel electrophoresis and analysed by immunoblotting with indicated antibodies. Commercially bought Erk2 and GST-GSK3 were used as positive controls. HI represents co-treatment with heregulin and IGF-1.

Although activation of TGF-β/Activin signalling is primarily determined by the receptor-mediated phosphorylation of Smad2/3 C-terminal SxS motif that induces their accumulation within the nucleus, it is increasingly apparent that the linker region of Smad2/3 serves a critical site through which their activity is regulated. This region contains multiple S/T residues, which are targeted by various S/T kinases stemming from other signalling pathways^24^,^25^,^26^,^27^,^28^. Phosphorylation of these residues alters the interaction of Smad2/3 with other proteins, which subsequently affects Smad2/3 stability, translocation and transcriptional activity^3^,^23^,^29^,^30^. Recruitment of Nedd4L to Smad2/3 was shown to be dependent on the phosphorylation status of their linker T220/T179 residue, which lies directly upstream of the PPXY-binding motif (Fig. 4a)^23^. Therefore, we anticipated that PI3K signalling might affect Smad2/3 degradation through altering the phosphorylation of this residue. Indeed, LY treatment significantly reduced the phosphorylation of T220/T179, irrespective of Smad2/3 activation, but had negligible effects on the other linker serine residues in both hESC and tumour cell lines (Fig. 4b–d; Supplementary Fig. 4a–e). This suggests that Smad2/3 linker threonine and serine residues are differentially regulated, with PI3K specifically modulating the phosphorylation of the T220/T179 linker residue. Our results also demonstrate that PI3K-mediated phosphorylation of this residue can occur in the absence of Smad2/3 activation (Fig. 5a,d), which raised the question as to whether Nedd4L binds to both active and inactive Smad2/3 or solely to the active form. To address this, PC3 cells, which retain high levels of PI3K signalling even after overnight starvation due to the presence of a PTEN mutation, were used for Nedd4L co-immunoprecipitation experiments upon treatment with AA±LY post starvation. This showed that Nedd4L recruitment and ubiquitination of Smad2/3 occurred most effectively only when both T220/T179 and SxS residues were phosphorylated (Fig. 4e), offering an explanatory mechanism by which Nedd4L selectively targets active Smad2/3 for ubiquitination and turnover.

We hence postulated that PI3K promotes phosphorylation of Smad2/3-T220/T179 and primes Smad2/3 for the binding of Nedd4L upon activation, resulting in increased ubiquitin-mediated Smad2/3 degradation, which acts to reduce the transcriptional activation of endoderm genes. If this is correct, mutagenesis of T220/T179 to a non-phosphorylatable residue should enhance the resistance of Smad2/3 to degradation independent of LY. To test this, we ectopically expressed wild-type (WT) or T220V mutant Smad2 in hESCs and PC3 cells. PC3 cells expressing Smad2-T220V showed higher Smad2/3 activation upon AA stimulation irrespective of whether LY was present (Fig. 4f) and correspondingly, hESCs expressing Smad2-T220V exhibited improved mesendoderm differentiation in response to AA alone (Fig. 4g). In further support of the view that Nedd4L-mediated degradation of active Smad2/3 accounts for the inhibitory effect of PI3K during Activin-induced DE differentiation, Nedd4L knockdown in PC3 cells resulted in increased Smad2/3 activation in the absence of LY (Fig. 4h; Supplementary Fig. 4f), while Nedd4L-deficient hESCs expressed endoderm genes at much higher levels upon AA stimulation (Fig. 4i). Taken together, PI3K signalling therefore inhibits Activin-induced DE differentiation by phosphorylating the T220/T179 of Smad2/3, enabling the recruitment of Nedd4L upon their activation, which leads to the degradation and termination of active Smad2/3.

### Smad2/3-pT220/pT179 is a direct effect of PI3K activity

Although many kinases including Erk (extracellular-signal-regulated kinases), p38 and CDK (cyclin-dependent kinase) have been shown to phosphorylate Smad2/3 linker residues^24^,^26^,^27^,^30^, inhibitors that specifically target these kinases were less effective in suppressing T220/T179 phosphorylation when compared with LY (Fig. 5a,b; Supplementary Fig. 5a). Notably, flavopiridol-mediated inhibition of CDKs only diminished the phosphorylation of the linker serine residues, whereas the T220/T179 residue was unaffected. Furthermore, PI3K-mediated phosphorylation of T220/T179 requires neither Smad2/3 activation nor nuclear localization, which thereby eliminates the direct involvement of CDK in causing this effect. Although active Erk can phosphorylate the T220/T179 residue, inhibition of PI3K elicited a greater reduction in T220/T179 phosphorylation than that of MAPK/Erk inhibition. In addition, LY treatment did not inhibit, but rather increased Erk activation under certain conditions (Fig. 5c; Supplementary Fig. 5a–c)^14^, which therefore excludes the possibility of LY-induced downregulation of T220/T179 phosphorylation being an Erk-related event. Furthermore, treatment of hESCs in RPMI base media along with heregulin and IGF-1 (HI), two potent stimulators of the PI3K pathway, increased T220/T179 phosphorylation (Fig. 5d), suggesting that this phosphorylation is regulated by the PI3K pathway itself independent of Erk and CDK activity.

Since Akt is one of the principal kinases acting downstream of PI3K, we next investigated whether Akt was responsible for T220/T179 phosphorylation by ectopically expressing constitutively active (Myr-Akt-WT), partially active (S473A) and inactive (K179M/T308A/S473A) Akt in Hep3B cells, using green fluorescent protein (GFP)-expressing Hep3B as controls. In comparison with the marked upregulation and reduction of T220/T179 phosphorylation observed in control cells upon heregulin and IGF-1 and LY treatment, respectively, expression of Akt possessing varying states of activation had negligible impact on T220/T179 phosphorylation (Fig. 5e), suggesting that Akt is not directly responsible for this phosphorylation. This was further confirmed by an *in vitro* kinase assay that demonstrated the inability of fully active Akt to phosphorylate this residue (Fig. 5f), which is consistent with previous findings^8^,^9^,^10^.

### mTORC2 induces the phosphorylation of Smad2/3-T220/T179

**Figure 6:**
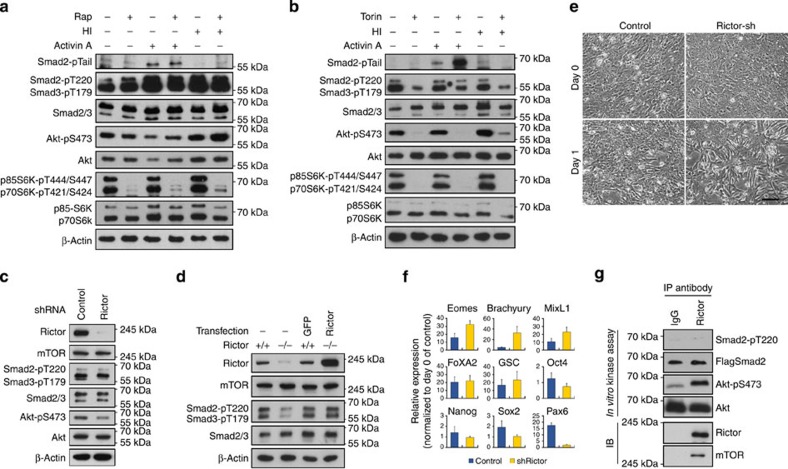
mTORC2 regulates Smad2/3 activity by modulating the phosphorylation of T220/T179 residue. (**a**) Effect of rapamycin on Smad2/3 signalling. PC3 cells were treated with rapamycin in the presence of Activin A or HI for 6 h and then harvested for immunoblot with indicated antibodies. (**b**) Effect of Torin treatments on Activin-Smad2/3 signalling. Immunoblot of PC3 cells treated similar as in **a** but replacing rapamycin with Torin-2 (Torin). (**c**,**d**) Reduction of Smad2/3-pT220/T179 in rictor-shRNA knockdown PC3 cells (**c**) and in rictor null MEF (**d**). Ectopic expression of human rictor in rictor null MEF reverted the levels of Smad2/3-pT220/T179. (**e**,**f**) Knockdown of rictor in hESCs accelerated morphological changes associated with DE specification (**e**) and increased expression of DE markers (**f**) in response to Activin A. Scale bar, 100 μm. hESCs in **f** were treated with Activin A for 2 days before the analysis and data represent mean±s.d. from at least six measurements of two independent experiments. (**g**) *In vitro* kinase assay of mTORC2 on Smad2-T220 phosphorylation. mTORC2 complexes were isolated by immunoprecipitation with rictor antibody and incubated with recombinant GST-Flag-Smad2 or inactive Akt in the presence of ATP. The protein mixtures were then resolved by SDS–polyacrylamide gel electrophoresis and analysed by immunoblot.

The mTOR branches of the PI3K signalling pathway acts to regulate cell growth, differentiation and metabolism. mTOR is the kinase component of two distinct multi-protein complexes, mTORC1 and mTORC2, which differ not only in their constituent components, but also in their response to rapamycin^31^. mTORC1 contains an adaptor protein, raptor, as a key subunit, while mTORC2 comprises rictor as its novel component; while mTORC1 activity is rapidly inhibited by rapamycin, mTORC2 is more resistant to acute rapamycin exposure^32^. To investigate whether mTOR complexes are involved in Smad2/3 regulation, PC3 cells were treated with AA in the presence of rapamycin or Torin-2 (henceforth Torin), an mTOR inhibitor that inhibits both complexes. While rapamycin showed no obvious effects on both Smad2 activation and T220/T179 phosphorylation (Fig. 6a), Torin treatment mimicked that of LY in prolonging Smad2 activation and inhibiting T220/T179 phosphorylation (Fig. 6b), thereby suggesting that mTORC2, rather than mTORC1, is involved in the regulation of Smad2/3 activity. In support of this notion, phosphorylation of T220/T179 was shown to be downregulated in rictor knockdown PC3 cells (Fig. 6c) and this reduction of pT220/pT179 appeared more evident in MEFs (mouse embryonic fibroblasts) that were derived from rictor null mice (Fig. 6d)^33^. The difference between rictor knockdown and rictor null cells could be due to the incomplete removal of rictor in the former as we have shown that PI3K/mTORC2-dependent phosphorylation of T220/179 is highly sensitive to any trace amount of PI3K/mTORC2 activity (Fig. 5d vs Supplementary Fig. 5b). Furthermore, this downregulation of T220/T179 phosphorylation in rictor null cells was fully rescued by the ectopic expression of human rictor (Fig. 6d), highlighting the important role mTORC2 plays in regulating Smad2/3-T220/T179 phosphorylation. Moreover, hESCs expressing rictor short hairpin RNA (shRNA) exhibited improved DE differentiation in response to AA treatment evidenced by rapid morphological changes and increased expression of DE markers, effects that are reminiscent of those observed upon AA–LY treatment (Fig. 6e,f). Therefore, these results suggest that mTORC2 plays an important role in modulating Activin-induced Smad2/3 activity by modifying the phosphorylation of the Smad2/3 linker T220/T179 residue. Intriguingly, immunoprecipitated mTORC2 was unable to phosphorylate Smad2-T220 in an *in vitro* kinase reaction (Fig. 6g), while inhibition of SGK1, one of the limited substrates identified to be regulated by mTORC2 (refs 34, 35), exhibited considerable reduction of both active and total Smad2/3 (Supplementary Fig. 6), effects that are completely different to that of either LY treatment or mTORC2 inhibition. Thus, the exact mechanisms by which mTORC2 regulates the phosphorylation of Smad2/3 linker residue await further elucidation.

### Suppression of mTORC2 leads to more efficient DE formation

**Figure 7:**
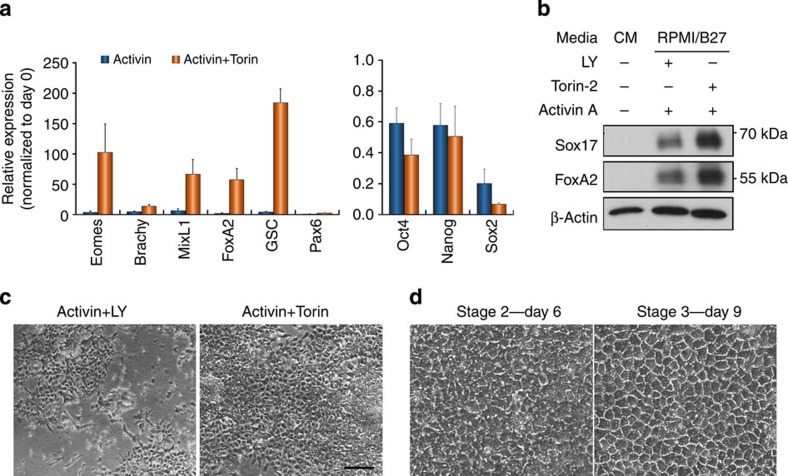
Inhibition of mTOR enhances DE differentiation of hESCs. (**a**) qRT–PCR showing gene expression in hESCs treated with Activin A±Torin for 2 days. Data represent mean±s.d. of six measurements from two independent experiments. (**b**) Immunoblot showing protein expression in hESCs treated as in **a**. (**c**) Phase-contrast images of hESCs treated for 3 days with Activin A together with either LY or Torin. Scale bar, 100 μm. (**d**) Phase-contrast images of the cells from **c** that were further differentiated by a hepatic differentiation protocol as shown in Fig. 1. Days of differentiation are indicated. Scale bar, 50 μm.

Consistent with the posited role for mTORC2 in regulating T220/T179 phosphorylation and inhibiting Smad2/3 activity, treatment of hESCs using a combination of AA and Torin yielded a substantial increase in the expression of endoderm genes (Fig. 7a,b), recapitulating the effects of AA–LY treatment, but with noticeably less cell death. Consequently, this led to both a more efficient DE differentiation and hepatocyte generation (Fig. 7c,d). Thus, the greater yield of DE cells in combination with the reduction in cytotoxicity makes AA–Torin a superior alternative to AA–LY treatment and provides compelling evidence to suggest that the mTORC2-mediated regulation of Smad2/3 linker phosphorylation is a critical mechanism accounting for the inhibitory effect of PI3K on Activin-induced DE differentiation.

## Discussion

In the present study, we have identified a novel and direct role for PI3K/mTORC2 signalling in regulating the phosphorylation status of the Smad2/3 linker T220/T179 residue. This phosphorylation permits the recruitment of the E3 ubiquitin ligase Nedd4L to Smad2/3 upon their activation, which subsequently promotes the ubiquitin-mediated proteasomal degradation of active Smad2/3. PI3K/mTORC2-mediated degradation of active Smad2/3 curtails the duration and intensity of the Activin-Smad2/3 signal, which ultimately leads to the attrition of Activin-induced DE differentiation in hESCs.

Our results are in line with previous findings, in that ubiquitin-mediated proteasomal degradation of active Smad2/3 plays an important role in the regulation of TGF-β/Smad2/3 activity^22^,^23^ and that the phosphorylation of Smad2/3 linker T220/T179 is critical for the recruitment of the E3 ubiquitin ligase Nedd4L^23^. However, we have found that the T220/T179 residue can be readily phosphorylated by PI3K/mTORC2 signalling in the absence of Smad2/3 activation and is thus independent of AA-induced CDK or Erk activation. Furthermore, this phosphorylation can only recruit Nedd4L efficiently upon Smad2/3 activation, thereby offering a mechanism by which preferential selectivity for active Smad2/3 degradation is established. In addition, TGF-β ligands are able to activate PI3K signalling through non-canonical means concurrently with the canonical activation of Smad2/3 (ref. 36), which may act as an internal regulative mechanism that prevents overactivation of Smad2/3. However, given that the majority of active Smad2/3 is still able to translocate into the nucleus despite the presence of T220/T179 phosphorylation (Supplementary Fig. 4e), this suggests that additional factors that interact with active Smad2/3 may hinder the recruitment of Nedd4L and prevent their degradation. Receptor-mediated activation of Smad2/3 has been well characterized for inducing conformational changes that enable the homo- and heterodimerization of R-Smads, as well as interaction with Smad4. However, whether or not such interactions between the Smad2/3 and other factors can act to impede the recruitment of Nedd4L awaits further investigation. In any case, despite only a small proportion of the activated R-Smads being targeted for degradation, this appears to be sufficient in impeding the proper formation of DE from hESCs. To definitively establish that Smad2/3 linker phosphorylation mediates the mTORC2-dependent inhibition of DE differentiation of hESCs, one would need to generate hESCs with knock-ins of linker phospho-deficient or phospho-mimetic mutants of Smad2/3.

**Figure 8:**
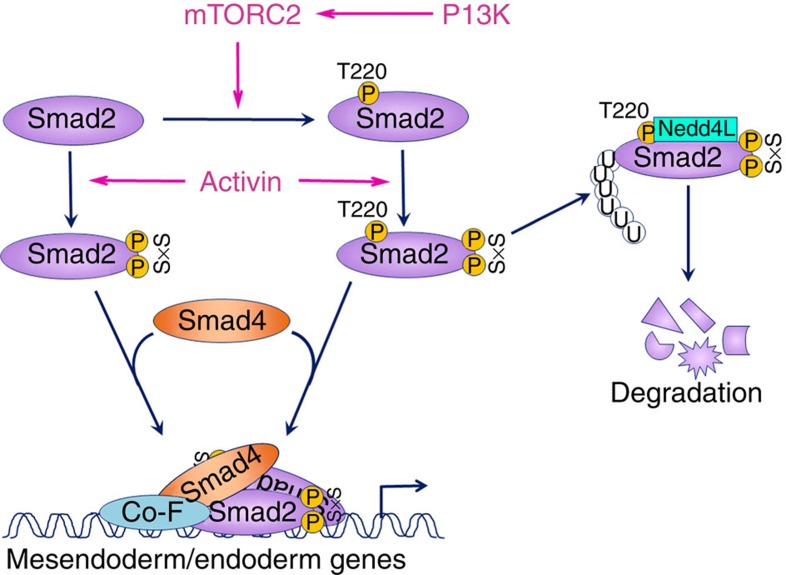
Model depicting a possible mechanism accounting for the inhibitory effect of PI3K/mTORC2 on the Activin-induced DE differentiation of hESCs. .

As shown in our experiments, AA–LY-induced enhancement of Smad2/3 activation in hESCs is only apparent at 3 h post treatment, despite the effectiveness of LY in reducing T220/T179 phosphorylation by 1 h of treatment. In explaining this discrepancy, it is probable that initially in the presence of abundant ligand (100 ng ml^−1^ AA) and persistent receptor activation^37^, the rate of Smad2/3 activation outpaces that of its Nedd4L-mediated degradation, which consequently obscures the effect of LY-induced downregulation of T220/T179 phosphorylation in prolonging Smad2/3 activation. However, as the ligand availability diminishes with increasing time, the proportion of the active Smad2/3 that is targeted for Nedd4L-mediated degradation becomes increasingly higher relative to the total active Smad2/3 pool, which ultimately promotes the rapid downregulation of Smad2/3 activity (Fig. 8). In support of this, the LY-dependent effect on active Smad2/3 was shown to be evident at 1 h post treatment when a lower dose of ligand (10 ng ml^−1^ AA) is applied or when receptor activation is blocked by SB431542. Putting this in a biological context, during embryonic development where TGF-β/Nodal/Activin are at physiological levels, this PI3K/mTOR/Nedd4L-mediated degradation mechanism may act as an internal thermostat that regulates the amplitude of Smad2/3 induction and thereby allowing proper readout of the morphogenic gradient *in vivo*.

One important finding of this study is the identification of mTORC2 as a modulator of TGF-β signalling, specifically by regulating the phosphorylation of the Smad2/3 linker T220/T179 residue. In assessing the contribution of the previously identified linker kinases, Erk1/2 and CDK^24^,^26^,^27^, we showed that when inhibited, neither could replicate the effects of PI3K inhibition. Furthermore, under certain conditions, LY-mediated inhibition of PI3K did not reduce, but rather enhanced the activation of Erk1/2. As such, enhancement of DE differentiation via downregulation T220/T179 phosphorylation and enhancement of Smad2/3 activity does not appear to be solely through an Erk/Wnt/β-catenin-mediated mechanism as outlined in a recent study^14^, but is rather through a more distinct and direct mechanism. The PI3K pathway bifurcates at many points and constitutes a large family of serine/threonine kinases of which the best characterized are the Akt and mTOR branches. Our data have shown that Akt does not affect phosphorylation of Smad2/3-T220/T179, which is consistent with previous findings^8^,^9^,^10^. In manipulating mTORC2 activity using both inhibitor-based and genetic means, we have identified mTORC2 as a key player in the regulation of the linker T220/T179 phosphorylation opposed to mTORC1. Although it has been reported that inhibition of mTORC1 by rapamycin can enhance the DE specification of hESCs^38^, chronic exposure of hESCs to rapamycin over several days will also inhibit mTORC2 activity^32^, which may ultimately be the reason for the reported improvement in DE specification. Furthermore, since mTORC2 activation is mediated by PI3K^39^, it is not surprising that the inhibition of PI3K signalling could lead to a cessation of mTORC2 kinase activity. Although mTORC2 immunoprecipitates could not phosphorylate the T220/T179 residue in an *in vitro* kinase reaction, this cannot completely exclude the possibility of mTORC2 being the direct kinase responsible for this phosphorylation, as the substrate recognition mechanisms of mTORC2 are still poorly defined^39^,^40^. Alternatively, mTORC2 may indeed act via indirect means through activation of downstream kinases that in turn regulate Smad2/3 linker phosphorylation; however, such kinases remain to be identified.

As a whole and to the best of our knowledge, we have for the first time identified a role for mTORC2 in mediating TGF-β/Activin signalling activities, and established a plausible mechanism through which mTORC2 activity directly impacts the Activin-induced DE differentiation of hESCs. Identification of mTORC2 as a key component of this mechanism not only provides new avenues through which hESC differentiation protocols can be improved, but also warrants the initiation of further studies into the impact of this mechanism on other TGF-β activities, adding to the growing repertoire of novel mTORC2 functions.

## Methods

### Cell culture and differentiation

H1 and H9 hESCs from WiCell were routinely cultured on Matrigel-coated plates in MEF conditioned medium (MEF-CM) supplemented with 10 ng ml^−1^ of bFGF^41^. DE differentiation was carried out as summarized in Fig. 1a^12^. When hESCs were grown to 70–80% confluency, MEF-CM was replaced with RPMI-1,640 supplemented with 1 × B27 (RPMI/B27 medium) containing Activin (100 ng ml^−1^, PeproTech) and LY294002 (20 μM day 1 and 10 μM day 2 and 3, Sigma) or Torin-2 (15 nM, Tocris) or wortmannin (300 nM, Sigma) for 2–3 days with daily medium change (Supplementary Fig. 1b). For hepatocyte differentiation, cells were further cultured in knockout-DMEM (KO-DMEM) containing 20% KO serum replacement, 1 mM glutamine, 1% non-essential amino acids, 0.1 mM β-mercaptoethanol (all from Life Technologies) with either DMSO (1%, Sigma) or BMP2 (20 ng ml^−1^, PeproTech) and bFGF (10 ng ml^−1^, R&D systems) for another 4 days, followed by 3–5 days culture in L15 medium supplemented with 8.3% foetal bovine serum (FBS), 8.3% tryptose phosphate broth, 10 μM hydrocortisone 21-hemisuccinate, 1 μM insulin (all from Sigma), 50 μg ml^−1^ ascorbic acid and 2 mM glutamine containing 10 ng ml^−1^ hepatocyte growth factor (PeproTech) and 20 ng ml^−1^ oncostatin M (R&D systems). In the experiments using various inhibitors, hESCs were cultured in RPMI/B27 medium containing Activin (100 ng ml^−1^) for 20 min following MEF-CM removal and 2 × PBS wash (Fig. 3a). The Activin-containing medium was then replaced with fresh RPMI/B27 containing the indicated inhibitors, and cells were harvested 1–6 h later as indicated. The following inhibitors were used: MG132 (10 μM) and okadaic acid (5 nM) from Merck/Millipore; SB431542 (10 μM) from Sigma; U0126 (10 μM) and SP600125 (500 nM) from Reagents Direct; SB203580 (20 μM) from R&D systems, flavopiridol (1 μM) and GDC-0941 (200 nM), both from Selleck Bio.

PC3 cells were kindly provided by Dr Kypta of Imperial College London. Hep3B and HEK293T tumour cell lines were obtained from ATCC (HB8064 and CRL-3216, respectively). Rictor null and corresponding control MEF were a gift from Professor Magnuson, VUMC, Tennessee^33^. PC3 cells were cultured in RPMI-1,640 medium containing 10% FBS. Hep3B, HEK293T and transgenic MEF cells were cultured in DMEM containing 10% FBS. All reagents for tumour cell cultures are from Sigma. Treatment of PC3 cells were performed by starving the cells overnight in RPMI-1,640 medium and then treating them for 1 h with Activin (10 ng ml^−1^)±LY (20 μM) or indicated factors, including rapamycin (100 nM), LongR^3^ IGF-1 (I, 200 ng ml^−1^, both from Sigma), Heregulin (H, 10 ng ml^−1^, PeproTech) and SGK inhibitor, GSK650394 (100 μM, Sigma).

All cell lines have been regularly checked for the absence of mycoplasma.

### Plasmids

Expression vectors for WT Smad2 (pCS2-Flag-Smad2, #14042), active Akt (pcDNA3 Myr-HA-Akt1, #9008) Akt triple mutant (pcDNA3-T7-Akt-K179M/T308A/S473A, #9031) and Rictor (pRK-myc-Rictor #11367) were obtained from Professor J. Massague^42^, Professor W. Sellers^43^ and Professor D. Sabatini^44^ through Addgene. Smad2 mutant T220V was generated by site-directed mutagenesis of pCS2-Flag-Smad2 using the Q5 Site-Directed Mutagenesis Kit (NEB) following the manufacturer's protocol, with the following oligonucleotides: forward primer 5′- GAGTAATTATATTCCAGAAGTGCCACCTCCTGGATATATC -3′ and reverse primer 5′- TGTGGCTCAATTCCTGCTGG -3′. The resulting sequences were validated by bidirectional sequencing. Partially active Akt (S473A) plasmid was generated by subcloning to replace the *Blp*I/*EcoR*I fragment of pcDNA3-Myr-HA-Akt1 containing S473 with A473 from pcDNA3-T7-Akt1-K179M/T308A/S473A. Lentivector containing shNedd4L was generated by cloning shNedd4L oligonucleotides into modified pLVTHM expressing puro-2A-GFP complementary DNA (cDNA)^45^. The sequence of shNedd4L was as previously published^23^: 5′- GCTAGACTGTGGATTGAGTttcaagagaACTCAATCCACAGTCTAGCtttttggaaa -3′. Lentivectors that contained two different shRNAs targeting Rictor within a pLKO.1 backbone were obtained from Addgene and used in combination to generate Rictor deficient lines. The sequences are as previously published^46^: 5′- ccggtCAGCCTTGAACTGTTTAActcgagTTAAACAGTTCAAGGCTGtttttg -3′ and 5′- ccggtACTTGTGAAGAATCGTATCTTctcgagAAGATACGATTCTTCACAAGTtttttg -3′. Lentivectors containing non-targeting shGFP was used to generate control cells. GST-Flag-Smad2/Smad2-T220V plasmids were generated by inserting NcoI/XhoI fragment containing Flag-Smad2 or Flag-Smad2-T220V from their respective expression vectors into the pGEX-6p2 vector (GE Healthcare).

### Transfection and lentiviral transduction

Hep3B cells and transgenic MEFs were grown to 70–90% confluency on the day of transfection. Akt or GFP expression plasmids were transfected using Lipofectamine LTX with PLUS reagent (Life Technologies) with modifications. In brief, for 1 × 10^6^ tumour cells, 1.6 μg of kit-purified plasmid DNA was suspended in 50 μl of OptiMEM supplemented with 1.6 μl of PLUS reagent. In another tube, 4 μl of Lipofectamine LTX reagent was resuspended in another 50 μl of OptiMEM. Both tubes were incubated at room temperature for 5 min before being mixed together and incubated for a further 25 min. During this time, target cells were trypsinized, counted and pelleted before being directly resuspended in the transfection mix and incubated for 15 min. The transfection process was stopped upon the addition of growth media and cells were allowed to plate down overnight in the appropriate culture vessel. Cells were typically split 1:2–1:3 following transfection. Reagents were scaled up accordingly depending on the number of cells to be transfected. For hESCs and MEFs, lipofection was conducted following the accompanying manufacturer's instructions with a DNA to Lipofectamine LTX ratio of 1:3 and 1:10, respectively. Transfected Hep3B cells were starved overnight the following day and then treated with the appropriate reagents for 1 h before being harvested for analysis. Transfected MEFs were allowed to recover and proliferate for 48 h before being harvested for immunoblot.

Lentiviruses were produced by transient transfection of HEK293T cells with a lentivector containing the desired transgenic cDNA or shRNA, as well as pCMVΔ8.91 helper and pVSV-G envelope plasmids using standard protocols^47^. hESCs were split 1:3 the day of transduction by being dissociated with accutase (Sigma) into single cells and resuspended in MEF-CM supplemented with 10 μM ROCKi (Y-27632 or ROCK inhibitor, Reagents Direct) to prevent apoptosis induced by the loss of cell–cell contact^48^. The cells were incubated overnight with concentrated viral particles and consequently became infected as they were plating down. Forty-eight hours post infection, cells were selected with puromycin (2 μg ml^−1^) and upon stable passaging, assessed for transgene expression or knockdown via immunoblot and quantitative reverse transcription (qRT–PCR), which was phenotypically assessed via DE differentiation.

### Quantitative reverse transcription–PCR (qRT–PCR)

Total RNAs were isolated from cells with TRI reagent (Sigma) and cDNA samples were synthesized with Protoscript II Reverse Transcriptase (NEB). qRT–PCR was performed in a DNA Engine Opticon (Bio-Rad) or Step One thermocycler (Applied BioSystems) using SYBR Green Jumpstart Taq Ready Mix (Sigma). Two housekeeping genes were used as normalizer. Two independent biological samples were used and multiple measurements were carried out for each treatment. Data were presented as mean±s.d. for six measurements. A list of primers used in this study are provided in Supplementary Table 1.

### Cytoplasmic/nuclear fractionation

Cells were harvested in cytoplasmic buffer (10 mM HEPES pH 7.9, 0.1 mM EDTA, 0.1 mM EGTA, 10 mM KCl and 0.5 mM dithiothreitol (DTT)) supplemented with phosphatase and protease inhibitors and allowed to swell on ice for 20 min after which Nonidet-P40 was added to a final concentration of 0.5%. Samples were quickly vortexed and centrifuged to pellet the cell nuclei. The supernatant was extracted and retained as the cytoplasmic fraction. The nuclear pellet was washed three times with cytoplasmic buffer to remove residual cytoplasmic contamination and resuspended in nuclear buffer (20 mM HEPES pH 7.9, 1 mM EDTA, 1 mM EGTA, 1 mM DTT and 0.4 M NaCl) before a final centrifugation at 15,000*g* to extract the nuclear fraction. Samples were quantified using bicinchoninic acid assay kit (Thermo Fisher Scientific) before being analysed by immunoblot.

### Antibodies

The following are the primary antibodies used in this study; indicated dilutions refer to use in immunoblotting, unless otherwise stated: anti-Akt (#9727, 1:2,000), pAkt-S473 (#4060, 1:2,000), pAkt-T308 (#9275, 1:1,000), Erk1/2 (#9102, 1:1,000), pErk1/2-T202/Y204 (#9106, 1:1,000), mTOR (#2972, 1:1,000), pNedd4L-S448 (#8063, 1:1,000), P70S6K (#9202, 1:1,000), pP70S6K-T421/S424 (#9204, 1:1,000), pGSK3α/β-S21/9 (#9331, 1:1,000), pRb-S780 (#9307, 1:1,000), pSAPK/JNK-T183/Y185 (#4668, 1:1,000), Smad2 (#3122, 1:1,000), Smad2/3 (#3102, 1:1,000), pSmad2-S245/250/255 (#3104, 1:1,000), pSmad2-S465/467 (#3122, 1:1,000), Smad4 (#9515, 1:1,000) and Ubiquitin (#3936, 1:1,000) were from Cell Signalling/New England Biolabs; anti-Brachyury (AF2085, 1:500, IF:1:50), CXCR4-PE (FAB170P, 1:25) and Sox17 (MAB1924, 1:500, IF:1:100) were from R&D systems; anti-CLIC4 (sc-130723, 1:500), Lamin B (sc-365962, 1:500) and PPM1A (sc-56956, 1:500) were from Santa Cruz; anti-AFP (A8452, 1:500), β-actin (A5316, 1:5,000), Flag (F1804, 1:1,000) and GST (G7781, 1:1,000) were from Sigma; anti-pSmad2/3-T220/179 (AP3675a, 1:500) and pSmad3-S423/425 (EP823Y, #04-1042, 1:1,000) were from Abgent and Millipore, respectively; anti-β-tubulin (ab6046, 1:1,000), FoxA2 (ab60721, 1:200) and Nedd4L (ab131167, 1:5,000) were from Abcam; anti-albumin (A0001, 1:500) was from DAKO and anti-Rictor (A300-459A, 1:5,000) was from Bethyl Labs.

Secondary antibodies for immunostaining used were as follows: Alexa Fluor goat anti-mouse IgG 488, 1:400, Alexa Fluor goat anti-mouse IgG 568, 1:400, Alexa Fluor goat anti-rabbit IgG 488, 1:400 and Alexa Fluor donkey anti-goat IgG 488, 1:400 were from Life Technologies. For immunoblotting: goat anti-rabbit IgG-HRP, 1:2,000, goat anti-mouse IgG-HRP, 1:5,000, donkey anti-goat IgG-HRP, 1:5,000 were from Santa Cruz and goat anti-rabbit or mouse IgG-HRP (light-chain specific), 1:10,000 were from Jackson ImmunoResearch Laboratories. Isotope controls: normal mouse IgG2a and normal rabbit IgG were from Santa Cruz.

### Immunostaining of hESCs

hESCs were split and cultured on Matrigel-coated Thermanox coverslips (Thermo Fisher Scientific) until the desired confluency was reached. Cells were then washed with Dulbecco's PBS (DPBS) and fixed for 10–20 min with 4% fresh paraformaldehyde solution. Excess paraformaldehyde was removed by washing three times with DPBS and the coverslips were then incubated with blocking/permeabilization buffer for 1 h followed by an overnight incubation with primary antibody at the appropriate dilution at 4 °C with tilting. The following day, coverslips were subjected to 10-min washes three times with DPBS, followed by a 40-min incubation with the appropriate fluorophore-conjugated secondary antibody in the dark. Coverslips were then subjected to two further 10 min washes with DPBS followed by a 10-min DPBS/4′,6 diamidino-2-phenylindole (DAPI) wash, with DAPI at a final concentration of 1 μg ml^−1^ to counterstain the nuclei. All washes were carried out in dark. Slides were mounted onto microscope slides using Mowiol 4–88 solution and allowed to dry overnight. Slides were visualized using a Leica SP5 II confocal fluorescent microscope typically at × 64 magnification.

### Immunoblotting

Cells were harvested using RIPA (radio immunoprecipitation assay) buffer (50 mM Tris-HCl, pH 8.0, 150 mM NaCl, 1% Nonidet-P40, 0.5% sodium deoxycholate and 0.5% SDS) supplemented with phosphatase (Na_3_VO_4_, 1:100, NaF, 1:200 or phosphatase inhibitor cocktail 2, Sigma, 10 μl ml^−1^) and protease inhibitors (phenylmethylsulphonyl fluoride in ethanol or protease inhibitor cocktail, Sigma). Proteins were purified by centrifugation at 15,000*g* at 4 °C for 20 min and then quantified using bicinchoninic acid assay kit (Thermo Fisher Scientific) and resolved on 7.5% Bis-Tris polyacrylamide gels, before being transferred onto polyvinylidene fluoride membranes via electroblotting. Blots were probed with primary antibody overnight followed by horseradish peroxidise (HRP)-conjugated secondary antibodies before being developed via enhanced chemiluminescent substrate and exposure onto CL-XPosure film (Thermo Fisher Scientific). Molecular weight markers (Thermo Fisher Scientific 11832124 or New England BioLab #7712) were run alongside all samples and values are as indicated in both the main and uncropped scans (Supplementary Fig. 7).

### Co-immunoprecipitation of Nedd4L with Smad2

Cells were harvested in HEPES lysis buffer (40 mM HEPES pH 7.4, 10 mM EDTA and 10% glycerol) supplemented with phosphatase and protease inhibitors as per immunoblotting. Cell lysis was induced mechanically by repeatedly passing lysate through a 23-G needle on ice. Protein within lysates were purified by centrifugation at 13,000*g* at 4 °C for 10 min and quantified, with 1 mg used in the subsequent immunoprecipitation with Smad2 antibody for 90 min at 4 °C with rotation. Immunocomplexes were isolated with the lysates using Protein-G conjugated Dynabeads (Life Technologies) through a further 1 h incubation under the same conditions. Beads were then washed 3 times with cold HEPES lysis buffer to remove nonspecific binding prior to protein elution with 2 × Laemmli buffer. Elutes were subjected to immunoblotting and the co-immunoprecipitation assessed using relevant primary antibodies.

### Smad2 ubiquitination assay

Cells were harvested in non-denaturing lysis buffer (20 mM Tris-HCl, pH 7.5, 150 mM NaCl, 1 mM EDTA, pH 8.0, 1 mM EGTA, pH 8.0 and 0.5% Nonidet-P40) supplemented with phosphatase and protease inhibitors as per immunoblotting. Lysates were additionally supplemented with 2 μM ubiquitin aldehyde (Boston Biochem) to prevent the action of deubiquitinating enzymes. Protein within lysates were purified and quantified, with 1 mg used in the subsequent immunoprecipitation with Smad2 antibody for 90 min at 4 °C with rotation. Immunocomplexes were isolated from the lysates using Protein-G conjugated Dynabeads through a further 1 h incubation under the same conditions. Beads were then washed 3 times with cold non-denaturing lysis buffer to remove nonspecific binding prior to protein elution with 2 × Laemmli buffer. Elutes were subjected to immunoblotting and the degree of ubiquitination was assessed using an anti-ubiquitin antibody.

### Generation of recombinant GST-Flag-Smad2/Smad2-T220V

pGEX-6p2 vectors containing Flag-Smad2/Smad2-T220V cDNAs were transformed into BL-21 *E. coli* strain. Upon ampicillin selection, single colonies were used to inoculate overnight cultures, which were then subsequently used to inoculate new cultures the following day. Expression of GST-Flag-Smad2/Smad2-T220V was induced in these cultures using 0.1 M isopropyl β-D-1-thiogalactopyranoside (Sigma) once they reached mid-log phase growth (OD_550–600_∼0.6–1.0). Proteins were harvested via centrifugation and sonication, followed by immunoprecipitation with glutathione-conjugated magnetic beads (Thermo Fisher Scientific). Recombinant proteins were eluted from the beads in 50 mM Tris-HCl containing 10 mM reduced glutathione. Proteins were then taken for immunoblotting with the relevant antibodies to check for successful expression and then used as substrate for subsequent kinase assays.

### Akt/Erk2 kinase assay

HEK293T or PC3 cells were harvested using non-denaturing lysis buffer (as per the Smad2 ubiquitination assay) to retain kinase activity and supplemented with phosphatase and protease inhibitors as per immunoblotting. Active Akt was immunoprecipitated using anti-pAkt (S473) antibody and was tested for kinase activity via incubation with recombinant GST-GSK3α/β fusion protein (NEB) as a substrate in kinase assay buffer (25 mM Tris-HCl, pH 7.5, 5 mM β-glycerophosphate, 2 mM DTT, 10 mM MgCl_2_ and 200 mM ATP) for 40 min at 30 °C. Kinase assay was repeated using GST-Flag-Smad2/Smad2-T220V as substrate and phosphorylation of the linker region was analysed via immunoblotting with the appropriate primary antibody. As a positive control, GST-Flag-Smad2/Smad2-T220V proteins were incubated with Erk2 kinase (NEB) in the supplied buffer supplemented with 200 mM ATP and assessed for phosphorylation via immunoblotting.

### mTORC2 kinase assay

The assay was carried out using previously described methods^46^ with the following modifications. HEK293T or PC3 cells were harvested in mTORC lysis buffer (40 mM HEPES pH 7.5, 120 mM NaCl, 1 mM EDTA, 10 mM Na_4_P_2_O_7_, 50 mM NaF and 0.5% Nonidet-P40) supplemented with phosphatase and protease inhibitors as per immunoblotting. Cells were gently lysed with rotation for 20 min at 4 °C after which 1 mg of total protein was used in the subsequent immunoprecipitation with Rictor antibody to selectively isolate mTORC2 over mTORC1. Immunoprecipitated complexes were washed 3 times with mTORC lysis buffer followed by washing with mTOR kinase assay buffer (25 mM HEPES, 100 mM potassium acetate, 1 mM MgCl_2_ and 500 mM ATP) twice to remove residual detergent. mTORC2 complexes were incubated with λ-phosphatase-treated His6-tagged-Akt1 (Millipore) protein or GST-Flag-Smad2/Smad2-T220V recombinant proteins in mTORC kinase assay buffer for 40 min at 37 °C. Reactions were stopped upon the addition of 2 × Laemmli buffer, and the phosphorylation on Akt-S473 or SmadT220 residues was assessed by immunoblotting with the appropriate primary antibodies.

### Luciferase assay

hESCs were split 1:6 into 12-well plates using the accutase/ROCKi method as above and allowed to reach 70–80% confluence prior to transfection. Both firefly pGL3-CAGA^12^-luc and renilla pRL-T7-renilla (Promega) plasmids were transfected at a ratio of 10:1 using Lipofectamine 2000 following the manufacturer's protocol. Forty-two hours post transfection, hESCs were differentiated for 6 h, harvested using accutase and dispensed into a 96-well luminometer plate. Luciferase assay was performed using the Dual-Glo luciferase assay kit (Promega) following the accompanying protocol. Both firefly and renilla luminescence was recorded using a Victor II luminometer (Perkin Elmer). Firefly luminescence readings were normalized with corresponding renilla luminescence readings to give representative luciferase expression values that are independent of transfection efficiency and cell number. Data presented are mean±s.d. from three independent transfection experiments.

## Additional information

**How to cite this article:** Yu, J. S. L. *et al*. PI3K/mTORC2 regulates TGF-β/Activin signalling by modulating Smad2/3 activity via linker phosphorylation. *Nat. Commun.* 6:7212 doi: 10.1038/ncomms8212 (2015).

## Supplementary Material

Supplementary Information Supplementary Figures 1-7 and Supplementary Table 1.
