# Supplementary material for: PI3K/mTORC2 regulates TGF-β/Activin signalling by modulating Smad2/3 activity via linker phosphorylation
Source: Nat Commun. 2015 May 22;6:7212. doi: 10.1038/ncomms8212 (PMC4455068; doi:10.1038/ncomms8212)
Supplement: Supplementary Information — Supplementary Figures 1-7 and Supplementary Table 1. [file ncomms8212-s1.pdf]

## Supplementary Information

# PI3K/mTORC2 regulates TGFβ/Activin signalling by modulating Smad2/3 activity via linker phosphorylation

Jason S.L. Yu,[1] Thamil Selvee Ramasamy,[1,2] Nick Murphy,[1] Marie Holt,[1] Rafal Czapiewski,[1] Shi-Khai Wei,[1] and Wei Cui [1,*]

### Supplementary Fig. 1

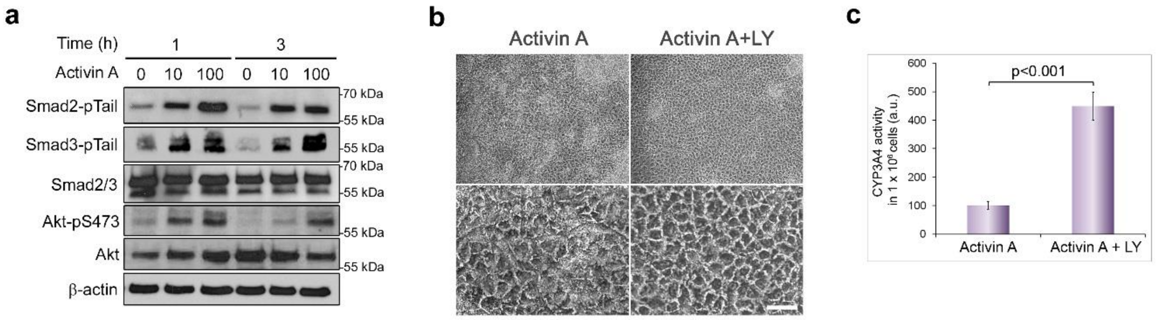


**Supplementary Figure 1** Inhibition of PI3K promotes DE and hepatocyte differentiation. (**a**) Activation of Smad2/3 in hESCs in response to various dosage of Activin A (ng $m^{-1}$ $l^{-1}$). (**b**) Phase-contrast images of hepatocyte-like cells differentiated from H9 hESCs that were treated with Activin ± LY for the first three days. Scale bar = 200 µm (upper panel) & 50 µm (low panel). (**c**) CYP3A4 activity in hepatocyte-like cells derived from hESCs treated with Activin ± LY during the first three days of differentiation. Standard derivation was calculated from three independent experiments.

**Supplementary Fig. 2**

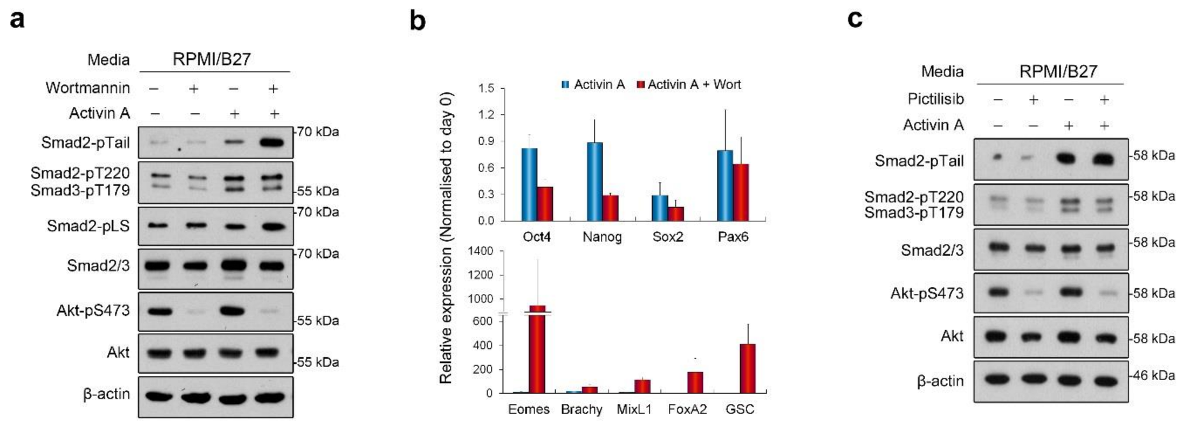


**Supplementary Figure 2** Effect of PI3K inhibitors on Activin-induced Smad2/3 signalling and DE differentiation in hESCs. (**a**) Immunoblot on cell lysates from hESCs treated with Activin A (100 ng $m^{-1}$ $l^{-1}$) ± wortmannin (300nM) for 6 hours. (**b**) Gene expression by RT-qPCR in hESCs differentiated with Activin A ± wortmannin for 2 days. Data are presented as mean ± SD from six measurements of two independent experiments. (**c**) Immunoblot on cell lysates from hESCs treated with Activin A (100 ng $m^{-1}$ $l^{-1}$) ± Pictilisib (GDC-0941, 200 nM) for 6 hours.

## Supplementary Fig. 3

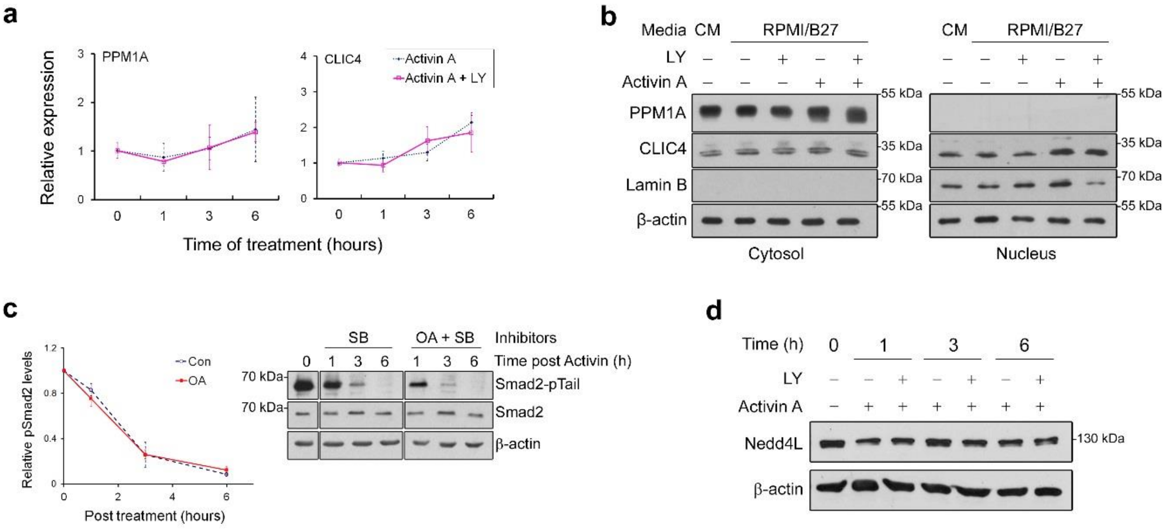


**Supplementary Figure 3** LY-induced enhancement of Smad2 activation is independent of phosphatase machineries. (**a**) Expression of PPM1A and CLIC4 mRNA by RT-qPCR in hESCs of indicted treatments. Data shows mean ± SD from three independent experiments. (**b**) Cytosolic and nuclear fractions of cell lysates from hESCs treated for 6 hours with indicated factors probed with PPM1A and CLIC4 antibodies. (**c**) Representative immunoblot (right) and quantifications (left) of activated Smad2 (Smad2-pTail) in hESCs treated as illustrated in Fig. 3a but with okadaic acid (OA). Graphs represent mean ± SD from three independent experiments. SB, SB431542. (**d**) LY treatment had no effect on Nedd4L expression in hESCs.

**Supplementary Fig. 4**

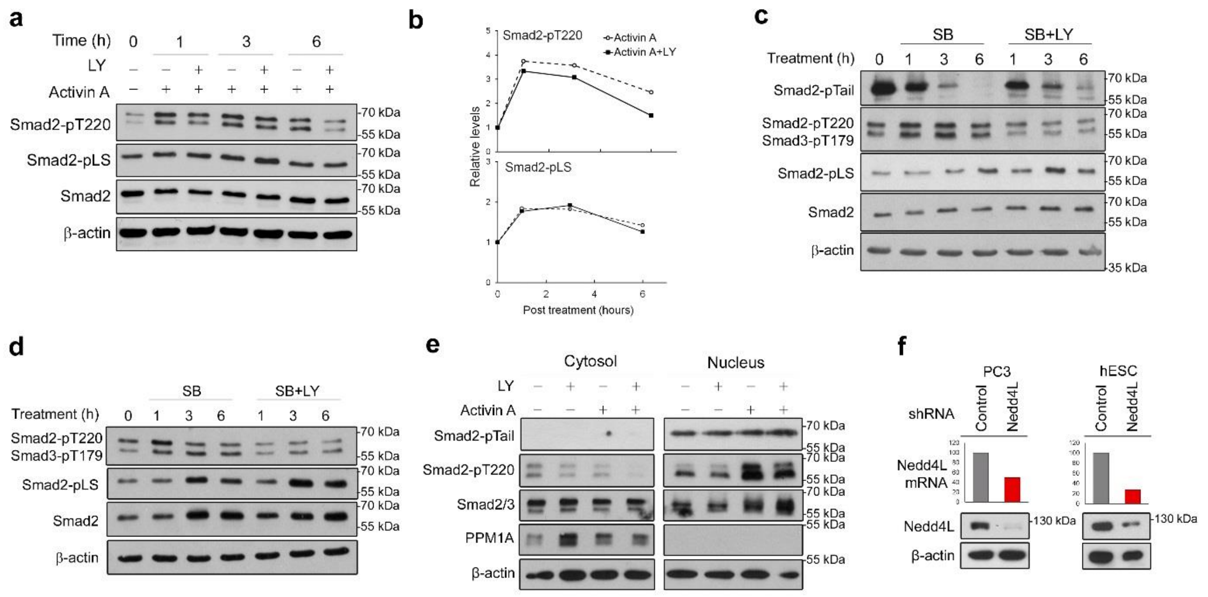


**Supplementary Figure 4** Smad2/3 linker phosphorylation. (**a-b**) Immunoblot (a) and quantification (b) of Smad2/3 linker phosphorylation in hESCs treated with Activin A ± LY for 1, 3 and 6 hours. (**c**) hESCs were pre-treated with Activin A for 20 minutes, then with SB and LY as indicated in Fig. 3a. Cell extracts were then analysed by immunoblot with indicated antibodies. (**d**) hESCs were treated similar to (c) but without initial Activin A stimulation. (**e**) Immunoblot of cytosolic and nuclear fractions from PC3 cells with 1 hour indicated treatment showing Smad2/3 phosphorylation at both C-terminal and linker residues. (**f**) qRT-PCR and immunoblot showing Nedd4L knockdown by shRNA in PC3 and hESCs.

## Supplementary Fig. 5

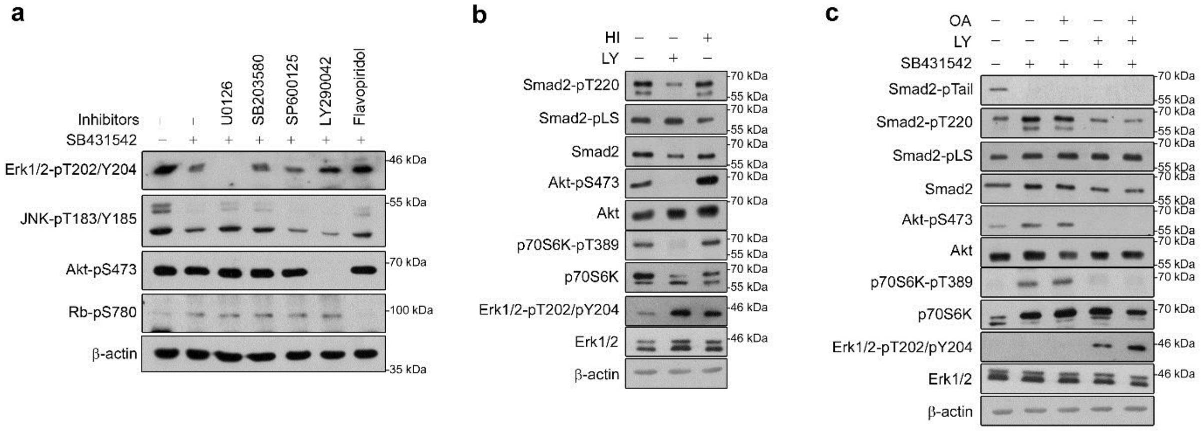


**Supplementary Figure 5** Effect of signalling pathways on Smad2/3 linker phosphorylation. (**a**) hESCs were pre-treated with Activin A for 20 minutes and then incubated with indicated inhibitors for 1 hour before analysed by immunoblot with indicated antibodies. (**b**) hESCs were cultured for 1 hour in RPMI/B27 medium with indicated factors and cell extracts were analysed by immunoblot with indicated antibodies. (**c**) hESCs were pre-treated with Activin A for 20 minutes in RPMI/B27 and then cultured for 1 hour with indicated factors. Cell extracts were analysed by immunoblotting.

## Supplementary Fig. 6

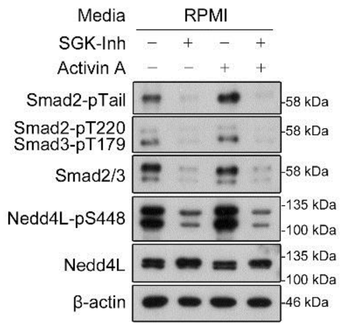


**Supplementary Figure 6** Effect of SGK1 on Smad2/3 signalling. PC3 cells were treated with SGK1 inhibitor, GSK650394 for 1 hour and cell lysates were analysed by immunoblotting with indicated antibodies.

**Supplementary Fig. 7-1**

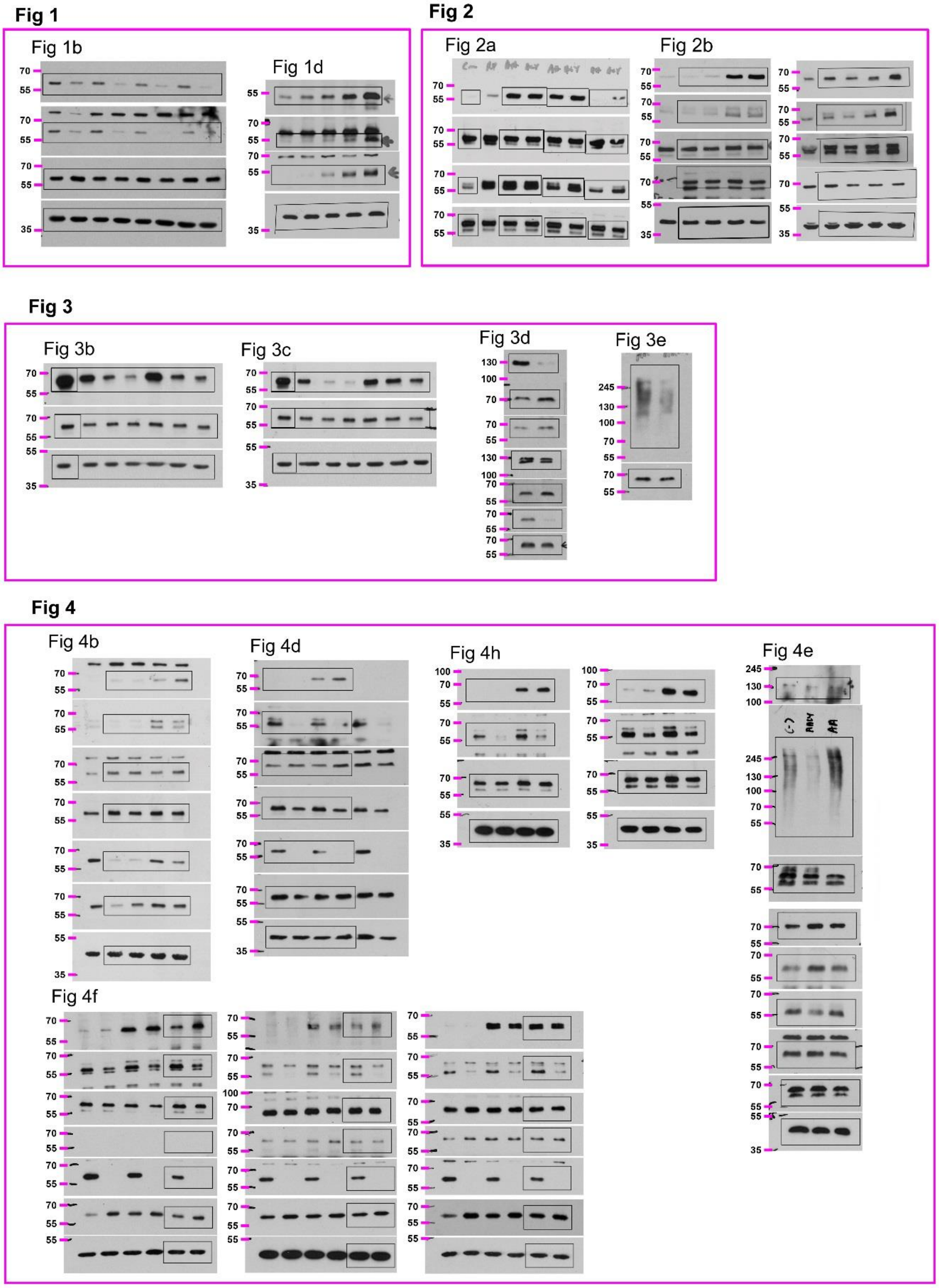

**Supplementary Fig. 7-2**

Fig 5

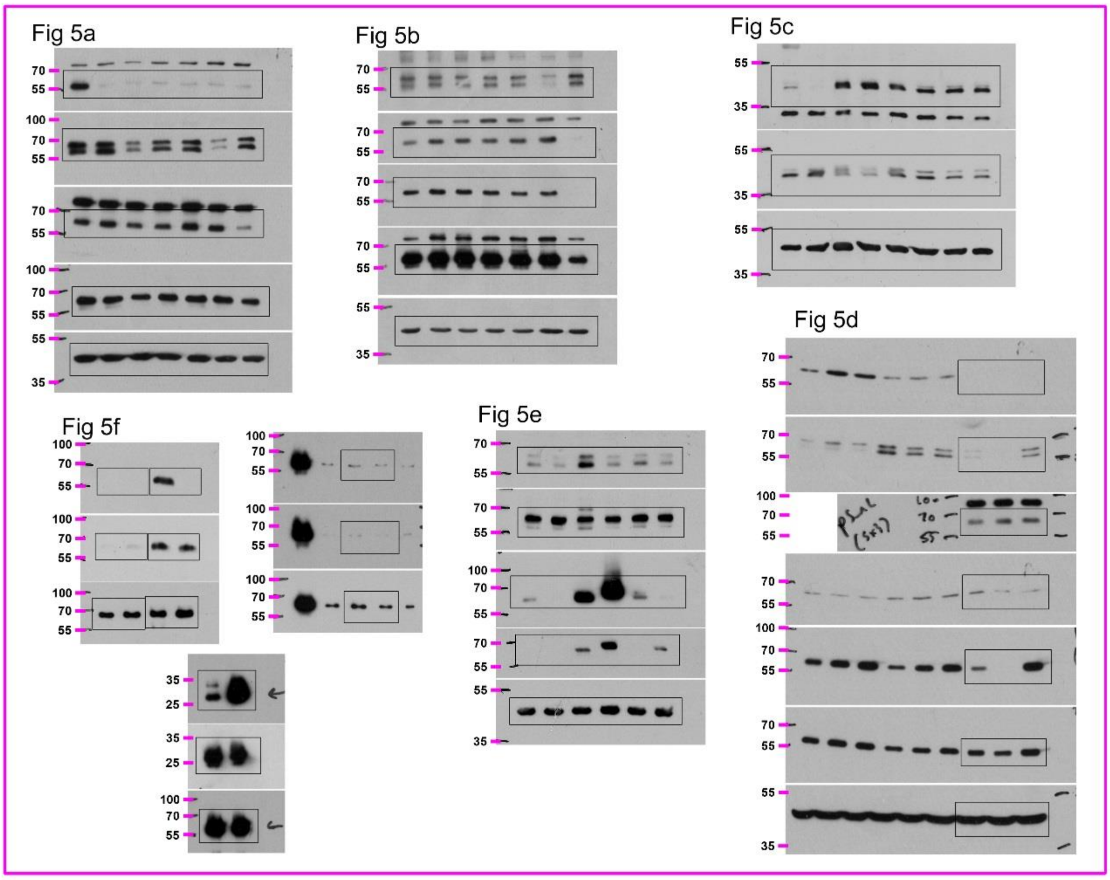


Fig 6

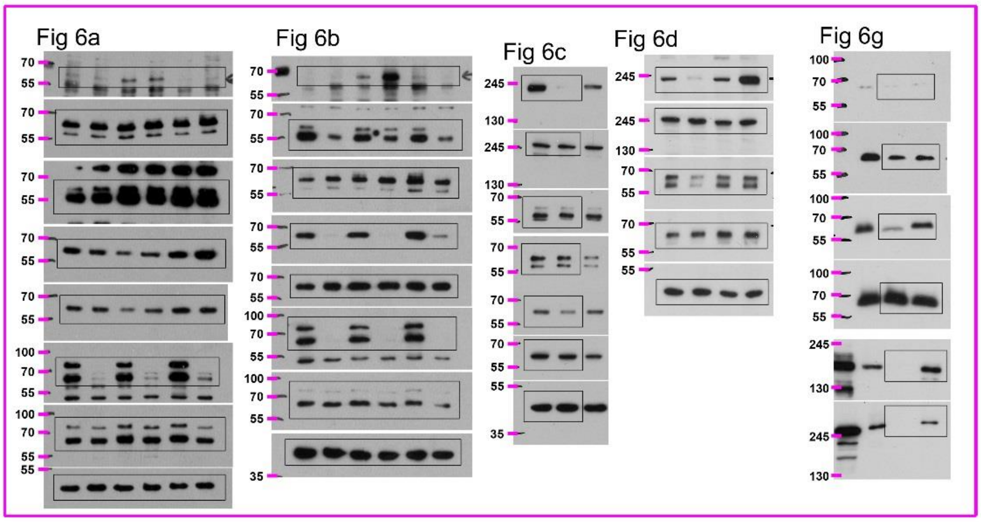


Fig 7

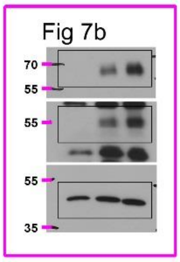

## Supplementary Fig. 7-3

### Supplementary Fig. 1

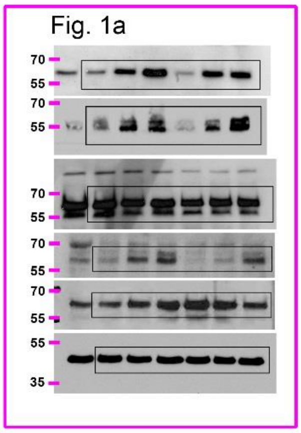


### Supplementary Fig. 2

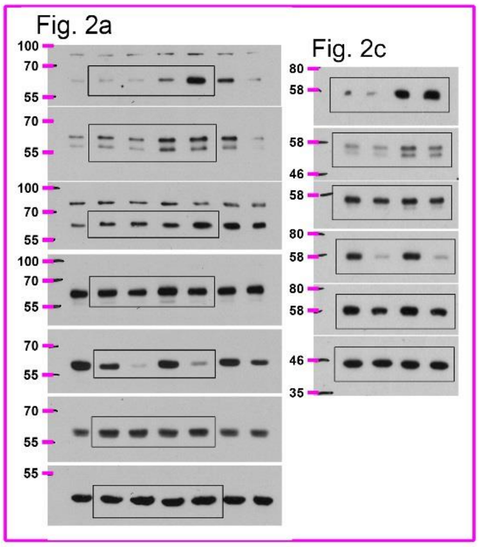


### Supplementary Fig. 4

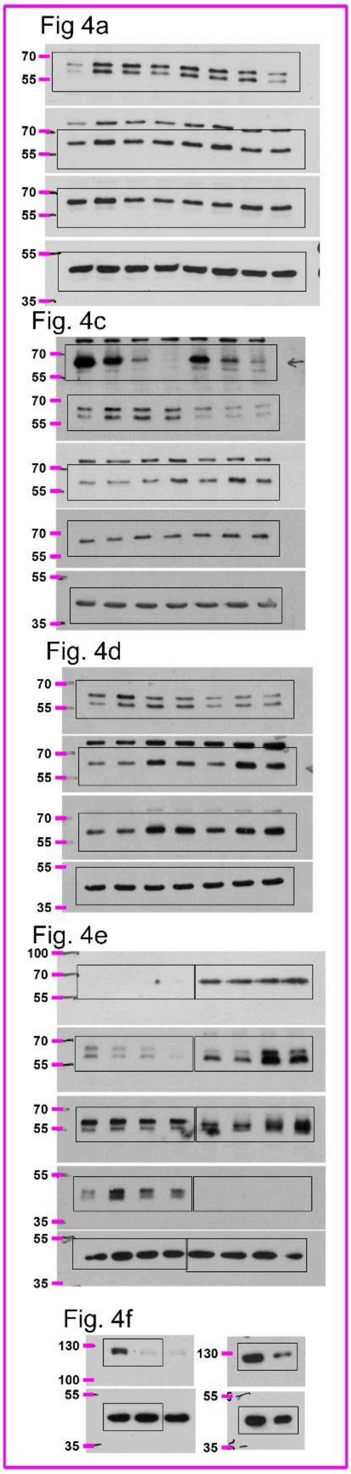


### Supplementary Fig. 3

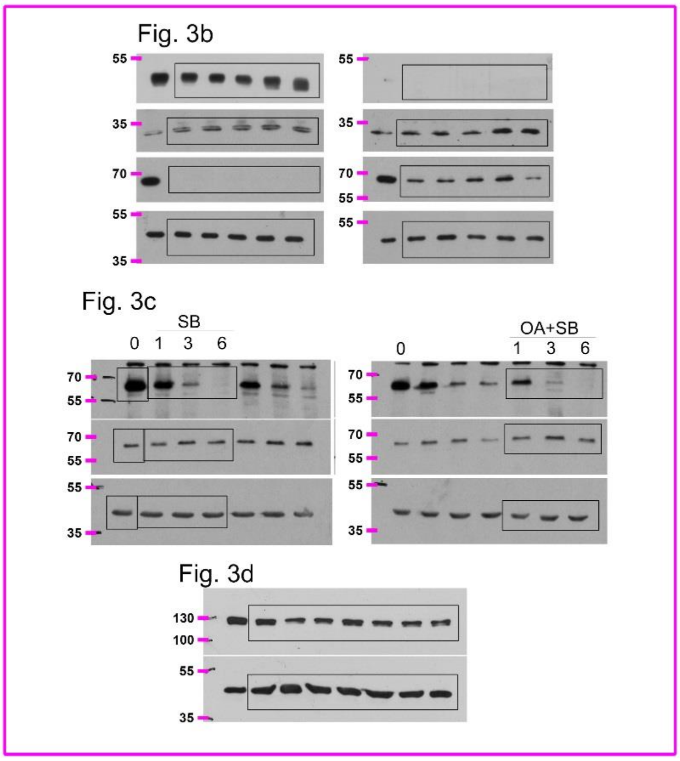

**Supplementary Fig. 7-4**

**Supplementary Fig. 5**

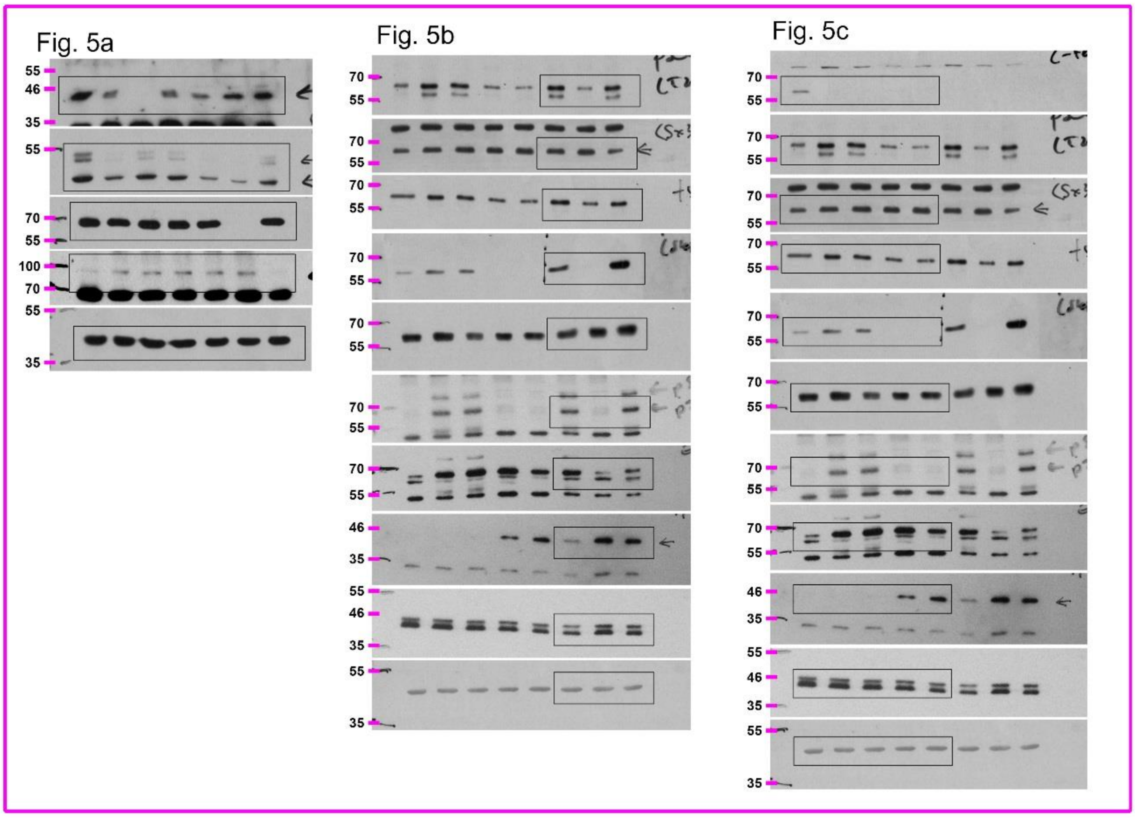


**Supplementary Fig. 6**

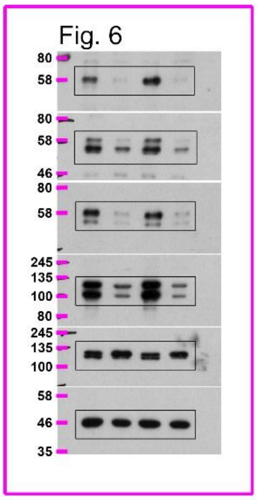


**Supplementary Figure 7** Original images of all immunoblots.

## Supplementary Table 1

**Supplementary Table 1:** List of Primers for qRT-PCR

| Gene | Forward primer (5'-3') | Reverse primers (5'-3') |
|---|---|---|
| Brachyury | TGCTTCCCTGAGACCCAGTT | GATCACTTCTTTCCTTTGCATCAAG |
| β-actin | TGTCTGGCGGCACCACCATG | AGGATGGAGCCGCCGATCCA |
| CLIC4 | TGAAAGCATAGGAAACTGCCC | GGTCAACAGTCGTCACACTAAA |
| E-cadherin | AGGAATTCTTGCTTTGCTAATTCTG | CGAAGAAACAGCAAGAGCAGC |
| Eomes | CGCCACCAAACTGAGATGAT | CACATTGTAGTGGGCAGTGG |
| Flag-Smad2 | GGACTACAAGGACGACGATGA | TCACTGCTTTCTCACACCACT |
| Fgf5 | CAGCACCAAAGGCTCAGCTT | CCTTGCTTCTAACCCATCATATCC |
| FoxA2 | GGGAGCGGTGAAGATGGA | TCATGTTGCTCACGGAGGAGTA |
| GAPDH | TCTGCTCCTCCTGTTCGACA | AAAAGCAGCCCTGGTGACC |
| GSC | GAGGAGAAAGTGGAGGTCTGGTT | CTCTGATGAGGACCGCTTCTG |
| MixL1 | CCGAGTCCAGGATCCAGGTA | CTCTGACGCCGAGACTTGG |
| Nanog | TGATTTGTGGGCCTGAAGAAAA | GAGGCATCTCAGCAGAAGACA |
| N-cadherin | CCCACACCCTGGAGACATTG | GCCGCTTTAAGGCCCTCA |
| Nedd4L | TCCAATGGTCCTCAGCTGTTTA | ATTTTCCACGGCCATGAGA |
| Oct4 | TCGAGAACCGAGTGAGAGGC | CACACTCGGACCACATCCTTC |
| Pax6 | TCCGTTGGAACTGATGGAGT | GTTGGTATCCGGGGACTTC |
| PPM1A | AGGGGCAGGGTAATGGGTT | GATCACAGCCGTATGTGCATC |
| RPL22 | TCGCTCACCTCCCTTTCTAA | TCACGGTGATCTTGCTCTTG |
| Smad2 | ATTCCAGAAACGCCACCTCC | GCTATTGAACACCAAAATGCAGG |
| Sox2 | GCCGAGTGGAAACTTTTGTCG | GCAGCGTGTACTTATCCTTCTT |
| Sox7 | GGCGCAGCAGAATCCAGA | CCACGACTTGCCCAGCAT |
| Sox17 | ACGCCGAGCTCAGCAAGAT | TCCACGTACGGCCTCTTCTG |
